# Blended Learning to Enhance Competencies Among Practicing Pharmacists: A Pre–Post Evaluation of the European Health Professionals’ and the DigitAl Team SkillS Advancement Project in Romania

**DOI:** 10.3390/pharmacy14030064

**Published:** 2026-04-24

**Authors:** Tünde Jurca, Andrei-Flavius Radu, Gabriela S. Bungau, Annamária Pallag, Anett Jolán Karetka, Octavia Gligor, Laura Graţiela Vicaş, Florin Bănică, Diana Teaha, Claudia Costea, Nóra Fazekas, Zoltán Cserháti, Ilie Cirstea, Tiberiu Sebastian Nemeth

**Affiliations:** 1 Doctoral School of Biological and Biomedical Sciences, University of Oradea, 410087 Oradea, Romania; tjurca@uoradea.ro (T.J.); apallag@uoradea.ro (A.P.); balasko.anettjolan@student.uoradea.ro (A.J.K.); banica@uoradea.ro (F.B.); cirstea.ilie@student.uoradea.ro (I.C.); snemeth@uoradea.ro (T.S.N.); 2 Department of Pharmacy, Faculty of Medicine and Pharmacy, University of Oradea, 410028 Oradea, Romania; lvicas@uoradea.ro; 3 Department of Psycho-Neuroscience and Recovery, Faculty of Medicine and Pharmacy, University of Oradea, 410073 Oradea, Romania; 4 Department of Preclinics, Faculty of Medicine and Pharmacy, University of Oradea, 410068 Oradea, Romania; octavia.gligor@uoradea.ro; 5 Bihor County College of Pharmacists, 410203 Oradea, Romania; dteaha@colegfarmbh.com (D.T.); ccostea@colegfarmbh.com (C.C.); 6 National Directorate General for Hospitals, H-1125 Budapest, Hungary; fazekas.nora@emk.semmelweis.hu (N.F.); cserhati.zoltan@emk.semmelweis.hu (Z.C.); 7 Health Services Management Training Centre, Semmelweis University, H-1125 Budapest, Hungary

**Keywords:** H-PASS, pharmacist training programme, healthcare, blended learning, pre–post evaluation, ceiling analysis, practicing pharmacist

## Abstract

The digital transformation of healthcare requires stronger digital competencies among pharmacists, yet evidence on the effectiveness of structured training remains scarce. This study examines the impact of a blended digital health training programme delivered to practicing pharmacists in Bihor County, Romania, as part of the Romanian pilot of the EU-funded European Health Professionals’ and the DigitAl team SkillS (H-PASS) project. A single-group pre–post educational design was applied to pharmacists from Bihor County, Romania, participating in a modular digital health training programme delivered between May and July 2025. A total of 84 pharmacists completed both pre-training and post-training self-reported competency assessments comprising 18 items across three modules: digital innovation and change management, communication and collaboration, and data management and digital literacy. Paired samples *t*-tests, Cohen’s d effect sizes, Cronbach’s alpha, moderator analyses, and ceiling effect analyses were conducted using Python-based statistical workflows. Statistically significant improvements were observed across all three modules (all *p* < 0.0001), with large effect sizes (d = 1.04–1.30). Post-training internal consistency increased substantially, with overall Cronbach’s alpha reaching 0.74. The greatest item-level gains were recorded in adaptive communication, cultural adaptation of care, and data protection ethics. No significant moderation effects were found for age, gender, or years of experience. Course satisfaction showed a moderate positive correlation with competency gains (r = 0.528), while perceived improvement was not significantly associated with observed score change. A ceiling effect indicated greater gains among participants with lower baseline competencies. The Romanian implementation of the H-PASS training programme was associated with improved self-reported digital health competencies among practicing pharmacists, high-lighting its potential as a scalable model for digital upskilling in healthcare.

## 1. Introduction

Digital transformation is increasingly shaping the structure and delivery of modern healthcare, driven by the integration of technologies such as information systems, telemedicine, cloud computing, and artificial intelligence. These innovations are reshaping healthcare delivery, supporting patient-centred models, improving efficiency, and enabling new educational approaches for health professionals [1,2].

As healthcare systems increasingly rely on digital infrastructures and data-driven practices, the development of digital competencies among healthcare professionals has emerged as a critical prerequisite for effective and sustainable digital health implementation [3]. Digital technologies are now embedded in medical and pharmaceutical practice, enabling access to patient information, remote communication, and digital disease management [4]. Consequently, health professionals require appropriate knowledge, skills, and competencies to effectively use these tools and adapt to the evolving digital healthcare environment [5].

Despite the growing urgency of digital transformation, recent evidence indicates that up to 80% of health professionals and students report feeling underprepared for the demands of eHealth and mHealth, highlighting persistent gaps in digital skills [6]. Moreover, the effective use of digital health technologies depends on health professionals having adequate digital knowledge, skills, and competencies. This highlights a persistent need for accessible, structured training programmes to support digital upskilling and reskilling across the health workforce [7].

Commission Delegated Directive (EU) 2024/782 updates the minimum training requirements for pharmacists, nurses, and dental practitioners to reflect scientific and technological progress across European Union (EU) health systems. For pharmacists, the revised framework explicitly integrates digital and information technology competencies into core education, alongside clinical pharmacy, pharmaceutical care, public health, and interprofessional collaboration. The Directive recognizes that modern pharmaceutical practice requires the practical application of digital tools and data-informed decision-making [8].

However, evidence from a mapping study of continuing education in digital skills across 25 EU member states highlights persistent national gaps and uneven implementation. Countries vary considerably in digital health policies, as well as in the organization and funding of training programmes. In many settings, continuing education remains fragmented across multiple providers (i.e., universities, employers, third parties, and authorities), with limited national coordination and inconsistent accreditation. In Romania, training initiatives are often employer-driven rather than embedded within a structured national framework, underscoring the need for targeted programmes [9].

In response to these structural gaps, the European Health Professionals’ and the DigitAl team SkillS advancement project (H-PASS) was developed to address the deficit in digital health competency training across European healthcare systems. H-PASS is a multi-country EU initiative funded by European Health and Digital Executive Agency, designed to strengthen workforce resilience by advancing transversal and digital competences among healthcare professionals through structured training programmes, blended learning methodologies, and pilot implementations across diverse European healthcare systems [10].

Within this European framework, Romania participated as one of the pilot implementation countries, adapting the standardized H-PASS training plan to the national pharmaceutical context through a blended-learning model combining asynchronous e-learning and synchronous trainer-facilitated sessions targeting core digital and transversal competencies [11].

Despite the growing body of literature on digital health education [12,13,14,15,16], empirical evaluations of structured training interventions targeting digital competencies among practicing pharmacists remain scarce, and pre–post evaluations with a range of statistical analysis remains limited. To the best of our knowledge, this represents one of the first studies to evaluate a structured digital health training intervention among practicing pharmacists in Romania in this manner. Within this European educational and policy context, evaluating the real-world implementations of structured digital training programmes becomes essential for understanding their practical impact on professional competence development. The present study represents the Romanian pilot evaluation within the H-PASS framework, aiming to evaluate pre–post changes in self-reported competency scores following participation in the training programme among practicing pharmacists from Bihor County, Romania, employing a pre–post design with analysis of competency score changes, internal consistency, moderating demographic factors, and the relationship between self-reported feedback and observed learning outcomes.

## 2. Materials and Methods

### 2.1. Study Design and Educational Intervention

The present study employed a single-group pre–post educational design to evaluate changes in self-reported digital competencies following participation of pharmacists in a modular digital health training programme delivered within the European H-PASS initiative between May and July 2025. The intervention followed a blended-learning format combining asynchronous e-learning activities with synchronous trainer-facilitated onsite sessions. For the present cohort, three competency domains were implemented: digital innovation and change management (Module 1), communication and collaboration in digital healthcare contexts (Module 2), and data management and digital literacy corresponding to H-PASS Module 4, selected as the optional core module in this cohort. For methodological clarity and consecutive numbering within the present analysis, modules are labelled 1–3, corresponding to H-PASS Modules 1, 2, and 4. The training targeted the development of self-reported digital health competencies through applied learning activities, individual and group exercises, and case-based scenarios. The evaluation focused on changes in participants’ self-reported competency profiles following exposure to the structured training intervention.

### 2.2. Data Collection and Assessment Instrument

A total of 90 pharmacists from Bihor County, Romania, enrolled in the H-PASS training programme. Participants were included in the final analytical sample if they completed both the pre-training (Welcome Survey) and post-training (Closing Survey) assessments, resulting in a final cohort of 84 participants with matched datasets.

Completers were defined as participants providing a full set of matched pre–post responses, and an attrition analysis was conducted to compare baseline characteristics between completers and non-completers to assess potential selection bias.

The assessment instrument consisted of a self-reported competence questionnaire rather than a knowledge test. Objective performance-based assessments were considered but were not feasible within this pilot implementation because the evaluation was embedded in a real-world continuing professional education programme for practicing pharmacists, where data collection needed to remain brief, low-burden, and compatible with routine programme delivery. In addition, no standardized performance-based instrument was available that mapped directly onto the three H-PASS modules implemented in this cohort within the project time-line. Accordingly, the present evaluation was designed to assess changes in perceived competence rather than directly observed task performance. Participants rated their agreement with competency-related statements on a 5-point Likert scale (1 = “Don’t agree”, 5 = “Completely agree”). The same competency items were administered in both the Welcome and Closing surveys to allow direct pre–post comparisons. The competency items included in the surveys were derived from predefined H-PASS learning outcomes covering knowledge, skills, and attitude across the implemented modules. The assessment instrument comprised 18 self-assessment items mapped to predefined learning outcomes, capturing both skill- and attitude-oriented competency domains across the implemented modules. The distribution of items across modules included four items for Module 1, eight items for Module 2, and six items for Module 3 (corresponding to H-PASS Module 4). Module 1 addressed digital innovation and change management, focusing on digital wellbeing, openness to new technologies, and the ability to plan and implement change processes in professional settings. Module 2 focused on communication and collaboration in digital healthcare contexts, including communication skills, teamwork, conflict management, and cultural competence in interactions with patients and colleagues. Module 3 covered data-related competencies, including the use of digital sources for data management, awareness of data quality and sources, data protection and safety, and the use of digital tools to support decision-making. A detailed mapping of survey items to competency domains is provided in Supplementary Table S1, while Supplementary Table S2 summarizes the structure and delivery of the training modules, and Supplementary Table S3 presents the post-training evaluation items included in the analysis.

In addition to the core competency self-assessment, the Closing Survey included a course evaluation section assessing participant satisfaction, perceived applicability to daily practice, perceived digital skill improvement, and confidence in digital teamwork. For the present analysis, four evaluation items were included: overall training satisfaction (E1), perceived applicability to daily work (E2), perceived digital skill improvement (E3), and perceived confidence in digital teamwork (E7). Evaluation items were analyzed descriptively and, where applicable, correlated with changes in self-reported competency scores to explore relationships between perceived educational value and observed changes in self-reported competency measures.

Raw survey data was exported and pre-processed using a custom pipeline. Data cleaning procedures included the standardization of categorical variables (e.g., gender, education level) and validation checks to ensure all Likert-scale responses fell within valid bounds (1–5). Composite scores for each module were calculated by averaging the constituent items.

Participation in the training evaluation was voluntary, and all survey responses were collected anonymously using participant-generated identifiers to ensure confidentiality. The study analyzed anonymized educational evaluation data collected within a professional training programme, and no identifiable personal information was processed. The study was conducted in accordance with the Declaration of Helsinki and received ethical approval from the Research Ethics Committee of the Faculty of Medicine and Pharmacy, University of Oradea (approval no. CEFMF/05, 31 May 2024).

### 2.3. Statistical Analysis

All data processing and statistical analyses were conducted using Python (version 3.12.3) [17]. Data manipulation and management were performed using the Pandas (v3.0.0) [18] and NumPy (v2.4.2) libraries [19], while SciPy (v1.17.0) [20] was used for hypothesis testing. Visualizations were generated using Matplotlib (v3.10.8) [21] and Seaborn (v0.13.2) [22].

#### 2.3.1. Reliability and Validity

The internal consistency of the assessment instrument was evaluated using Cronbach’s alpha. Reliability coefficients were calculated separately for each curriculum-defined module and for the full 18-item instrument at both pre- and post-intervention time points. Given that the items were mapped to predefined learning outcomes and included a limited number of items per module, these analyses were interpreted as descriptive indicators of internal consistency rather than as confirmation of a psychometrically validated latent scale structure. To our knowledge, this questionnaire had not previously been validated as an independent instrument for measuring digital competence among practicing pharmacists. For this reason, Cronbach’s alpha was used cautiously as a descriptive reliability index only. Negative alpha values at baseline were interpreted as indicating very weak average inter-item association within some curriculum-defined module groupings, consistent with heterogeneous domains and the small number of items per module, rather than as evidence of a scoring error or reverse coding problem. Exploratory validity evidence was additionally examined through Spearman rank correlations between changes in self-reported competency scores and self-reported measures of satisfaction and applicability.

#### 2.3.2. Effectiveness and Group Comparisons

Differences between pre- and post-training scores were primarily assessed using paired-samples *t*-tests at the module level. Normality of the paired pre–post difference scores was assessed using the Shapiro–Wilk test, supplemented by visual inspection of Q–Q plots, before selecting between parametric and non-parametric analyses. Normality of the paired pre–post difference scores was evaluated at the module level and for the overall composite score using the Shapiro–Wilk test. All four distributions showed statistically significant departures from normality (Module 1: W = 0.936, *p* < 0.001; Module 2: W = 0.947, *p* = 0.002; Module 3: W = 0.951, *p* = 0.003; Overall: W = 0.911, *p* < 0.001), and visual inspection of Q–Q plots (Supplementary Figure S1) indicated moderate deviations but no severe outliers. Despite these departures, paired-samples *t*-tests were retained as the primary analysis because parametric tests are robust to moderate violations of normality and ordinal measurement level, even with modest sample sizes [23], and the present outcomes are composite scores averaged across multiple Likert-type items, which further approximate continuous distributions. This approach is consistent with current recommendations for analyzing summated Likert-type scales in health education research [24]. Moreover, the departures observed in Q–Q plots were moderate rather than extreme, with no evidence of heavy tails or influential outliers. To confirm inferential robustness, Wilcoxon signed-rank tests were performed as non-parametric sensitivity analyses at both the module and item levels, and yielded identical inferential conclusions for all 22 comparisons (Supplementary Table S4). The complete concordance between parametric and non-parametric results indicates that the inferential conclusions are not sensitive to distributional assumptions in this dataset. Item-level analysis effect sizes (Cohen’s d) with 95% confidence intervals were calculated for each competency item to quantify the magnitude of improvement. Benjamini–Hochberg false discovery rate (FDR) correction was applied to control for multiple comparisons across the 18 items (α = 0.05). Benjamini–Hochberg false discovery rate (FDR) correction was applied to control for multiple comparisons across the 18 item-level tests (α = 0.05). The three module-level comparisons were treated as pre-planned primary analyses and were not subjected to additional multiplicity correction, consistent with recommendations that small families of a priori hypothesis tests do not require adjustment when the tests correspond to distinct, pre-specified research questions. An exploratory attrition analysis was performed to screen for potential selection bias by comparing baseline characteristics of completers and non-completers. The Mann–Whitney U test was used to compare baseline competency scores, while Chi-square tests were used to assess demographic differences in attrition rates. Given the small number of non-completers (*n* = 6), these comparisons were treated as descriptive and interpreted cautiously.

#### 2.3.3. Moderator and Convergence Analysis

A moderator analysis was conducted to determine if demographic factors (age, gender, experience) influenced training outcomes. Non-parametric tests were selected for moderator comparisons because the subgroup sizes were unequal and the change-score distributions did not meet parametric assumptions. Kruskal–Wallis H tests were used for multi-group comparisons (age groups, experience categories) and Mann–Whitney U tests for two-group comparisons (gender). Effect sizes were quantified using eta-squared (η^2^ = H/(*n* − 1) for Kruskal–Wallis; η^2^ = Z^2^/*n* for Mann–Whitney U), where values below 0.01 are considered negligible, 0.01–0.06 small, and above 0.06 medium. Results were synthesized into a heatmap for visual inspection, available in the Supplementary Material.

Finally, to investigate the relationship between baseline proficiency and learning outcomes, a ceiling effect analysis was performed using Pearson correlation coefficients. This examined the relationship between pre-training scores and the magnitude of change, testing the hypothesis that the training narrowed the perceived competency gap between participants with different baseline levels.

## 3. Results

The final analytical cohort consisted of 84 pharmacists with diverse demographic data, characterized by a high proportion of female professionals and a diverse range of experience levels (Table 1). The sample was predominantly female (*n* = 75, 89.3%), with male participants representing 10.7% (*n* = 9) of the cohort. The age distribution was relatively balanced across mid-career and senior brackets: the modal age group was 45–54 years (*n* = 28, 33.3%), followed by the 25–34 and 35–44 age groups (each *n* = 26, 31%). Participants in the early-career 18–24 group (1.2%) and the senior 55–64 group (3.6%) represented smaller segments of the population.

The cohort demonstrated significant professional maturity, with 65.5% of participants having more than 10 years of experience. Specifically, 41.7% (*n* = 35) reported 11–20 years of experience, while 23.8% (*n* = 20) reported over 20 years. Professionals with under 10 years of experience comprised 34.5% (*n* = 29) of the sample. Most participants held a bachelor’s degree (*n* = 72, 85.7%). Advanced degrees were also represented, including master’s degrees (*n* = 2, 2.4%) and doctorates (*n* = 10, 11.9%), reflecting the high level of academic qualification within the Romanian pharmaceutical sector.

Regarding the professional setting, the majority of participants practiced in urban areas (*n* = 68, 81.0%), while 13.1% (*n* = 11) were active in rural pharmacies. This distribution allows for exploratory comparisons between urban and rural practice contexts.

**Figure 1:**
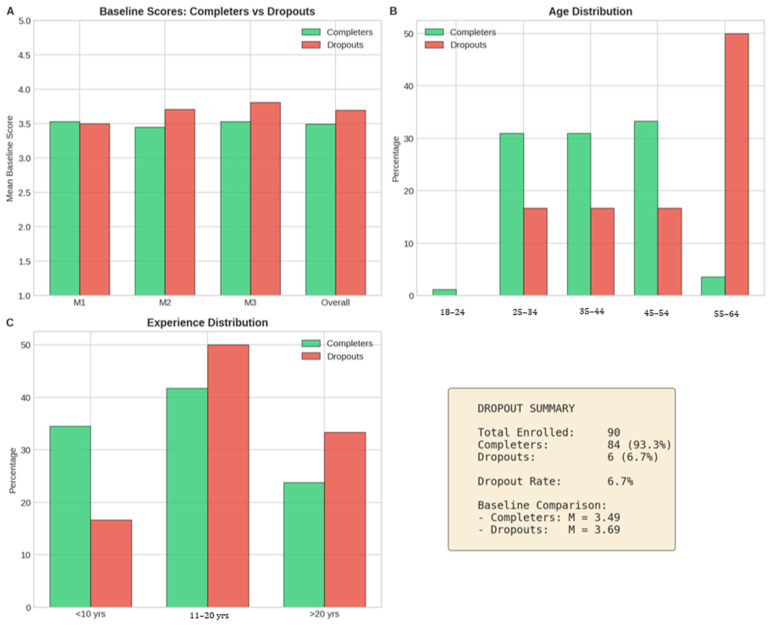
Exploratory attrition analysis comparing completers (*n* = 84) and non-completers (*n* = 6) among pharmacists enrolled in the Romanian H-PASS training programme. (**A**) Baseline mean self-reported competency scores for Modules 1–3 and the overall score. (**B**) Age-group distribution in the two groups. (**C**) Years-of-professional-experience distribution in the two groups. The summary panel reports cohort size, retention, and mean overall baseline score by completion status. Percentages are shown for demographic distributions.

Of the 90 professionals initially enrolled in the programme, 84 provided complete pre- and post-training datasets, yielding a retention rate of 93.3%. An exploratory attrition analysis (Figure 1) was conducted to compare baseline characteristics between completers (*n* = 84) and non-completers (*n* = 6). Non-completers showed higher baseline competency scores than completers (M = 3.69, SD = 0.27 vs. M = 3.49, SD = 0.24, Mann–Whitney U test, *p* = 0.03). However, given the very small number of non-completers, this finding should be interpreted cautiously, as the comparison was underpowered and the estimate may be unstable. No statistically significant differences were observed for gender, education level, or years of experience. Although these results raise the possibility that attrition was not entirely random, they do not support firm conclusions about systematic differences between completers and non-completers.

The internal consistency analysis showed very low baseline reliability for the curriculum-defined module scores, with Cronbach’s alpha values ranging from −0.12 to 0.11. Specifically, baseline alpha values were 0.02 for Module 1, 0.11 for Module 2, and −0.12 for Module 3. Internal consistency improved after the intervention, reaching 0.31 for Module 1, 0.60 for Module 2, and 0.53 for Module 3. When the instrument was examined as a complete 18-item measure, Cronbach’s alpha was −0.22 at baseline and 0.74 post-training. This pattern indicates that the predefined module groupings showed weak internal consistency at baseline and only modest improvement at the subscale level after training, whereas the full post-training instrument demonstrated acceptable internal consistency. Given the limited number of items per module and the heterogeneous competency domains represented, the module-level alpha coefficients should be interpreted cautiously as descriptive indicators rather than evidence of strong psychometric subscales. The reliability results are presented in Supplementary Figure S2.

**Figure 2:**
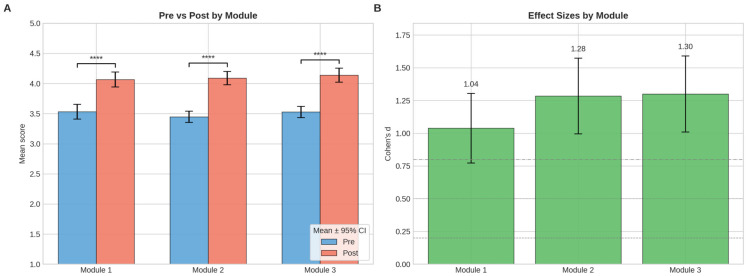
Module-level training effectiveness. (**A**) Mean pre-training and post-training module scores for Modules 1–3, shown as mean ± 95% confidence intervals. Brackets indicate paired pre/post comparisons; significance markers denote the corresponding *p*-value levels (**** *p* < 0.0001). (**B**) Module-level effect sizes (Cohen’s d) with 95% confidence intervals. Positive values indicate improvement following training.

Paired-samples *t*-tests showed significant improvements across all three modules from pre-training to post-training, as shown in Figure 2A. Module 1 increased from 3.53 to 4.07, with t = 9.52 and *p* < 0.0001. Module 2 increased from 3.45 to 4.09, with t = 11.76 and *p* < 0.0001. Module 3 increased from 3.53 to 4.14, with t = 11.91 and *p* < 0.0001. As shown in Figure 2B, effect sizes were large for all domains, with Cohen’s d of 1.04 (95% CI 0.77–1.30) for Module 1, 1.28 (95% CI 0.99–1.57) for Module 2, and 1.30 (95% CI 1.01–1.59) for Module 3.

**Figure 3:**
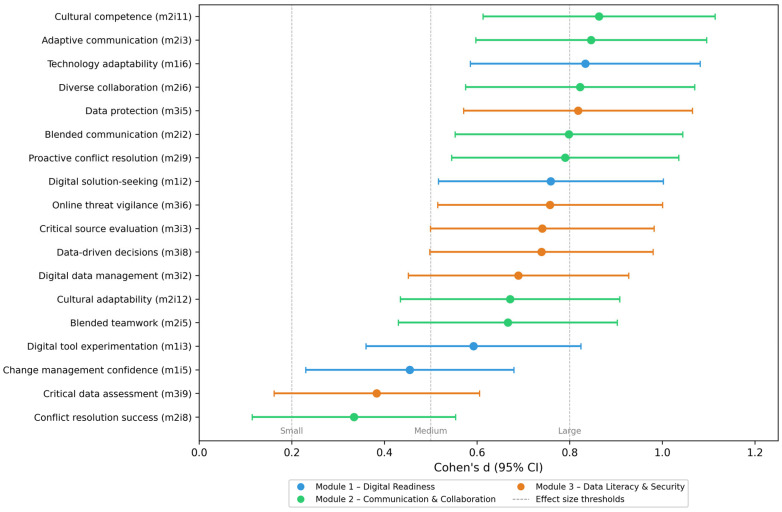
Item-level effect sizes (Cohen’s d) for the 18 self-reported competency indicators, sorted by magnitude (*n* = 84). Error bars represent 95% confidence intervals. Dashed vertical lines indicate conventional thresholds for small (0.2), medium (0.5), and large (0.8) effects. All 18 items reached statistical significance after Benjamini–Hochberg FDR correction (α = 0.05).

Figure 3 illustrates the effect sizes (Cohen’s d) with 95% confidence intervals for all 18 competency indicators, arranged in descending order of magnitude, revealing statistically significant growth across every skill assessed (*p* < 0.001) for all items after Benjamini–Hochberg correction. While improvements were universal, the largest effect sizes were observed for cultural competence (m2i11, d = 0.86), adaptive communication (m2i3, d = 0.85), and data protection (m3i5, d = 0.82), whereas more modest gains were found for conflict resolution (m2i8, d = 0.33) and critical data assessment (m3i9, d = 0.38). This pattern may suggest that the training was most effective for competencies directly addressed through structured content delivery, while skills requiring sustained interpersonal practice showed smaller, though still significant, improvements.

A moderator analysis was conducted to examine whether training-associated competency gains differed by age, gender, or years of professional experience. No significant differences were observed across age groups (*p* = 0.25), gender (*p* = 0.73), or experience groups (*p* = 0.67), and effect sizes were uniformly small (η^2^ < 0.05 across all categories; Supplementary Figure S3). These results suggest that demographic subgroup membership did not meaningfully moderate competency gains in this sample. However, this absence of significant moderation should not be interpreted as evidence of equivalence: the study was not powered for subgroup comparisons, and the unequal group sizes (e.g., 75 females vs. 9 males) limited statistical sensitivity to detect meaningful differences.

**Figure 4:**
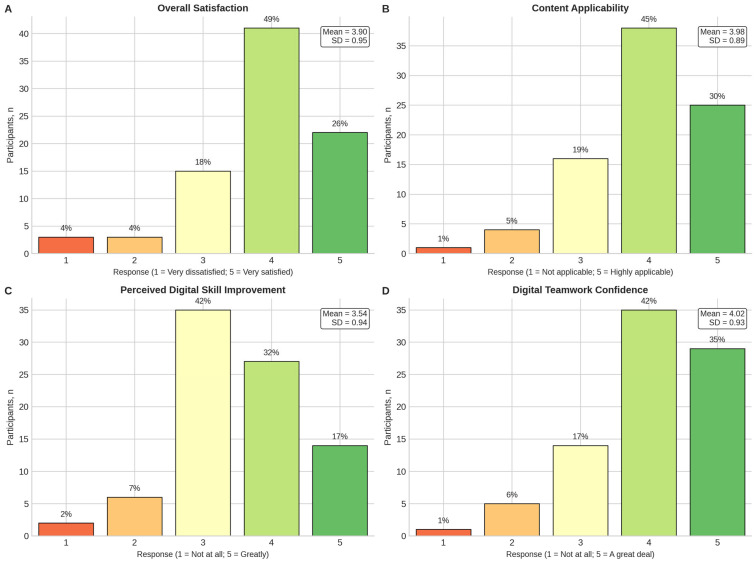
Distribution of post-training course-evaluation responses among participants with complete data (*n* = 84). (**A**) Overall satisfaction. (**B**) Content applicability to daily work. (**C**) Perceived digital skill improvement. (**D**) Confidence in digital teamwork. Responses were recorded on 5-point scales. For panel A, 1 = very dissatisfied and 5 = very satisfied. For panel B, 1 = not applicable and 5 = highly applicable. For panel C, 1 = not at all and 5 = greatly. For panel D, 1 = not at all and 5 = a great deal. Percentages above bars indicate the proportion of participants selecting each response category. Insets show the mean and standard deviation for each item.

Feedback collected post-training indicates a highly positive reception of the curriculum. As illustrated in Figure 4, the vast majority of participants rated the training as directly applicable to their professional duties (M = 3.98, SD = 0.89). Overall satisfaction levels were similarly high (M = 3.90, SD = 0.95), with 75% of the cohort reporting that they were either “satisfied” or “very satisfied” with the programme. The distribution of responses reveals a clear negative skew, with ratings clustered heavily at the upper end of the scale, suggesting that the training content effectively met the diverse needs of the pharmacist cohort. Furthermore, a Spearman rank correlation analysis demonstrated a significant positive relationship between competency gains and reported satisfaction. This finding suggests that participants who exhibited greater observed increases in self-reported competency scores were also those who perceived higher value in the training intervention.

To explore the relationship between participant feedback and measured changes in self-reported competencies, we analyzed the association between subjective survey responses (perceived improvement and course satisfaction) and observed changes in competency scores (post-test minus pre-test). Spearman’s rank correlation coefficient was calculated for each association (*n* = 84).

**Figure 5:**
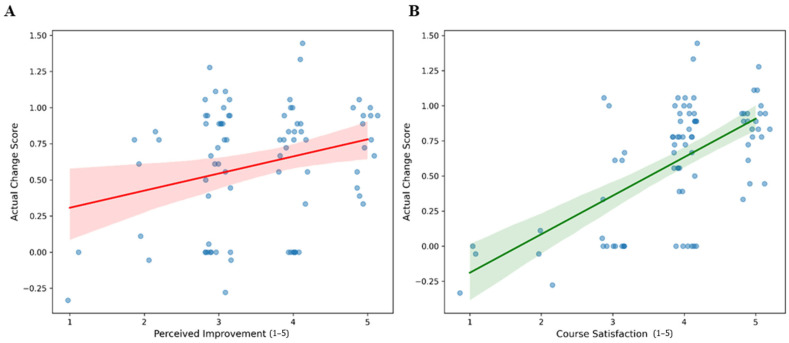
Associations between subjective post-training ratings and observed change in overall self-reported competency score (post-training minus pre-training) among participants with complete data (*n* = 84). (**A**) Relationship between perceived digital skill improvement and observed overall score change. (**B**) Relationship between overall course satisfaction and observed overall score change. In panel A, perceived digital skill improvement was rated on a 5-point scale from 1 = not at all to 5 = greatly. In panel B, overall course satisfaction was rated on a 5-point scale from 1 = very dissatisfied to 5 = very satisfied. Points represent individual participants. Solid lines indicate fitted linear trends, and shaded areas represent 95% confidence intervals.

Figure 5 (left panel) illustrates the association between self-reported skill development and observed changes in self-reported competency scores. The analysis revealed a weak positive correlation between participants’ perceived improvement and their actual score change (r = 0.195). However, this association was not statistically significant (*p* = 0.075), suggesting that participants’ perceived improvement ratings showed high variability relative to observed competency score change. The scatter plot demonstrates high variance, with several participants reporting maximum perceived improvement (5 out of 5) despite negligible or negative observed competency score changes.

In contrast, Figure 5 (right panel) demonstrates a statistically significant, moderate positive correlation between overall course satisfaction and observed competency score change (r = 0.528, *p* < 0.001). The regression line indicates that higher levels of course satisfaction were consistently associated with greater gains in competency scores.

**Figure 6:**
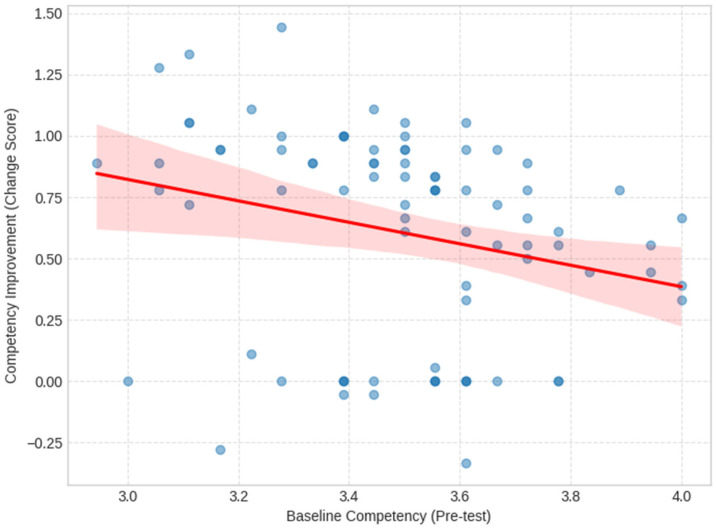
Ceiling-effect analysis showing the association between baseline overall self-reported competency score and observed change in overall score (post-training minus pre-training) among participants with complete data (*n* = 84). Each point represents one participant. The solid line indicates the fitted linear regression, and the shaded area represents the 95% confidence interval. The negative association is consistent with a ceiling effect, whereby participants with higher baseline scores had less room for improvement.

A ceiling effect analysis was performed to investigate the relationship between baseline proficiency and training outcomes. As illustrated in Figure 6, a significant negative correlation was observed between pre-training scores and the magnitude of improvement (r = −0.25, *p* = 0.02). This inverse relationship identifies a ‘ceiling effect,’ where participants with high initial competency had less room for growth, while those with lower baseline scores demonstrated the most substantial gains. Practically, this suggests the H-PASS curriculum was highly effective at narrowing the perceived competency gap across baseline levels, accelerating the learning curve for novices to ensure a more uniform standard of digital capability across the cohort.

## 4. Discussion

The primary objective of this study was to evaluate pre–post changes in self-reported digital competencies following participation in the H-PASS training programme among Romanian pharmacists. The results indicate substantial positive changes across digital literacy, communication, and data management domains after the intervention. However, because the study used a single-group pre–post design without a control group, these findings should be interpreted as evidence of positive change associated with participation rather than definitive proof that the intervention alone caused the observed improvements.

From an educational evaluation perspective, the observed improvements in participant satisfaction and self-reported competency scores may be interpreted within broader training evaluation frameworks that distinguish between learner reaction and learning outcomes, reflecting immediate post-training educational impact [25]. The results demonstrate statistically significant and practically large improvements across all three implemented modules, with effect sizes ranging from d = 1.04 to d = 1.30. These findings suggest that the Romanian pharmacist H-PASS training programme was associated with substantial positive pre–post changes, although these should be interpreted cautiously given the absence of a control group [26].

The blended learning format adopted by the H-PASS training plan, combining asynchronous e-learning with synchronous onsite sessions, aligns with widely described blended education models in health professional training. Evidence from systematic reviews and meta-analyses has reported improved knowledge outcomes compared with traditional learning approaches [27].

In an EU mapping study covering 25 member states, Romania and thirteen other countries were reported to lack comprehensive national regulation or a responsible authority coordinating continuing education in digital skills for healthcare professionals, highlighting fragmented approaches across Europe. Countries such as Slovenia, Spain, Austria, and France demonstrated stronger national strategies, coordination mechanisms, or structured accreditation systems for digital skills education. Training activities were primarily organized locally by educational institutions or employers, with accreditation often provided through professional continuing education credits rather than nationally coordinated digital skills programmes [9].

EU health workforce shows a digital-skills deficit, with 80% reporting insufficient eHealth/mHealth training, 30–70% lacking needed skills, and persistent gaps in comprehensive programmes, advanced-topic coverage and in-service training opportunities [7]. The present study represents one of the first empirical evaluations of the H-PASS training in a national professional cohort, providing early evidence for programme effectiveness in Romania, where the European H-PASS curriculum was adapted to the pharmaceutical practice context and delivered as a blended digital competency training programme for practicing pharmacists.

A noteworthy finding of this study relates to the internal consistency of the self-assessment instrument. These findings reinforce that the instrument should be interpreted primarily as a curriculum-aligned educational evaluation tool with content grounded in H-PASS learning outcomes, rather than as a fully validated psychometric scale designed to measure a single underlying construct. At baseline, Cronbach’s alpha values for the curriculum-defined module scores were very low, ranging from −0.12 to 0.11, and the alpha for the full 18-item instrument was also negative. After the intervention, internal consistency improved, with post-training alpha values of 0.31 for Module 1, 0.60 for Module 2, 0.53 for Module 3, and 0.74 for the full instrument. These results were confirmed after rechecking the dataset and were not explained by out-of-range values, zero-variance items, or an obvious reverse-coding error. Rather, they suggest that before training, self-reported competencies across the predefined domains were heterogeneous and only weakly interrelated. This pattern aligns with broader observations in digital health competence research. For example, Jarva et al. (2024) identified distinct digital health competence profiles among healthcare professionals and reported substantial variability across competence domains at baseline [28].

Accordingly, the module scores should be interpreted cautiously as curriculum-aligned domain summaries rather than as strongly internally consistent psychometric subscales. The acceptable post-training alpha observed for the full 18-item instrument suggests that the intervention may have increased the overall coherence of participants’ self-perceived digital competencies when the instrument is considered globally. However, the subscale-level reliability results indicate that the predefined modules do not function as highly homogeneous scales, which limits strong psychometric interpretation at the module level. This distinction is important when interpreting the observed training gains and supports the decision to emphasize the overall competency pattern alongside the curriculum-based domain analyses. These observations are consistent with a recent systematic review examining digital competency education among healthcare professionals, which report that educational interventions frequently improve knowledge, skills, self-efficacy, and confidence domains following training exposure [15]. They also align with the observation by Mainz et al. (2024) that existing digital competency measurement instruments often emphasize technical skills and knowledge and may not fully capture the broader methodological, social, and personal dimensions of digital competence [29].

The largest item-level effect sizes were observed for cultural competence (d = 0.86), adaptive communication (d = 0.85), and data protection (d = 0.82), whereas more modest gains were found for conflict resolution (d = 0.33) and critical data assessment (d = 0.38). This pattern may suggest that the training was most effective for competencies directly addressed through structured content delivery, while skills requiring sustained interpersonal practice showed smaller, though still significant, improvements. In contrast, previous evidence suggests that digital literacy development in pharmacy has historically lacked structured frameworks and clearly defined baseline competencies. A systematic review by MacLure and Stewart reported limited integration of digital literacy into pharmacy training and highlighted substantial unmet educational needs among pharmacists, reflecting a field where technology adoption has often outpaced workforce upskilling [30].

The relationship between participant feedback and measured score change provides a more nuanced view of training response. Overall satisfaction appeared to track more closely with observed improvement than participants’ own ratings of skill improvement, suggesting that positive engagement with the programme may be associated with stronger learning gains, whereas self-appraisal of improvement may be less well calibrated in this context. This pattern supports caution when interpreting self-reported improvement as a stand-alone outcome and aligns with work suggesting that confidence, self-efficacy, and competence do not always evolve in parallel [31,32]. It also reinforces the value of complementing self-report measures with more structured or performance-based assessments in future evaluations.

Furthermore, the ceiling analysis provides empirical evidence that the H-PASS training programme acted as a ‘leveler’ for professional capability. The significant negative correlation between baseline proficiency and improvement magnitude confirms a strong narrowing of the skills gap. While more proficient participants encountered a ‘ceiling effect’, the rapid acceleration of the learning curve for novices ensured that the cohort moved toward a more uniform standard of digital literacy. When combined with the fact that neither age nor years of experience hindered progress, these results provide evidence that challenges assumptions associated with the ‘digital native’ paradigm. It demonstrates that with a structured, equitable pedagogical approach, senior professionals can achieve parity with their younger counterparts, ensuring that the digital shift does not leave experienced clinicians behind.

The correlation analysis between subjective feedback and objective outcomes offered nuanced insights into professional self-assessment. Moreover, there was no statistically significant correlation between participants’ perceived improvement and their actual score change. The high variance in this relationship suggests that pharmacists may struggle to accurately judge their own learning curves in the digital domain, potentially due to the Dunning-Kruger effect [33] or a lack of prior benchmarks. Conversely, course satisfaction was a strong predictor of objective success (r = 0.528), indicating that engagement and positive reception of the material are key drivers of competency acquisition [34]. In line with Mainz et al., this dissociation may indicate that self-reported improvement reflects evolving confidence and self-efficacy alongside competence development, highlighting the value of complementing subjective feedback with more structured, performance-based assessment approaches [35].

The lack of a significant correlation between perceived improvement and objective score changes suggest a notable ‘metacognitive gap’ within the cohort. While participants were highly satisfied, their inability to accurately calibrate their own learning progress indicates that digital competency in pharmacy is often pre-reflective at the baseline. This aligns with the fragmented knowledge frameworks observed in the pre-training internal consistency analysis. Consequently, these findings argue against relying solely on self-reported surveys to evaluate digital health interventions. Without objective pre- and post-testing, the actual efficacy of such programmes may be masked by participant enthusiasm or a lack of established professional benchmarks.

Finally, the ceiling analysis highlighted a significant negative correlation between baseline scores and improvement magnitude. This “ceiling effect” indicates that while high-performing participants had less room to grow, the training effectively accelerated the learning curve for novices. Attrition analysis suggested that non-completers reported somewhat higher baseline competency scores; however, because only six participants were lost to follow-up, this pattern should be treated as tentative and not overinterpreted. Ultimately, the training succeeded in its goal of narrowing the skills gap and ensuring a uniform standard of capability across the profession. While satisfaction alone does not guarantee learning, evidence from blended learning research suggests that positive learner experience and motivation may support engagement and persistence, factors associated with improved knowledge outcomes in health education [26].

While the results are promising, several limitations must be acknowledged to contextualize the findings. First, the final cohort consisted of 84 pharmacists, representing a relatively robust and practice-relevant sample of healthcare professionals, although generalizability to the broader national population remains limited. Moreover, the attrition analysis comparing completers (*n* = 84) and non-completers (*n* = 6) was exploratory in nature. With only six dropouts, statistical power was limited and effect estimates should be regarded as tentative rather than definitive. This generalizability is further constrained by a geographic bias, as participants were predominantly located in urban areas (81%), meaning the specific digital infrastructure limitations faced by rural pharmacists may be underrepresented. Because participation in the training and evaluation was voluntary, self-selection bias cannot be excluded, and the cohort may have included pharmacists who were more motivated to engage with digital upskilling than the wider professional population. Regarding methodology, the study utilized a single-group pre–post design without a control group. Consequently, we cannot exclude the possibility that some of the observed improvements were influenced by external factors, such as secular trends, concurrent digital exposures, or repeated measurement effects. Additionally, because the primary outcomes relied on self-reported competency measures and post-training evaluations, the findings may also be affected by social desirability, acquiescence, or expectancy effects. Self-reported measures may also overestimate improvement, as participants’ ratings can reflect increased confidence, satisfaction, or perceived preparedness rather than objectively demonstrated performance. This is particularly relevant in short-term post-training evaluations, where positive response tendencies may inflate estimated gains. Therefore, the observed changes should be interpreted as improvements in perceived competence associated with programme participation rather than as direct evidence of behavioural or task-based proficiency. Although objective assessments such as standardized case-based testing, observed performance tasks, or practical simulations would have strengthened the evaluation, these approaches were not feasible in the present pilot because of time and implementation constraints within a voluntary, practice-based training programme. Because no comparison group, objective performance-based assessment, or negative-control survey items were included, the extent of these influences cannot be determined in the present study. Future research should incorporate controlled designs, such as comparison-group or stepped-wedge approaches, objective performance-based assessments, negative-control survey items, and longitudinal follow-up.

## 5. Conclusions

The present study provides empirical evidence of positive pre–post changes following participation in the H-PASS blended digital health training programme among practicing pharmacists in Bihor County, Romania. Statistically significant improvements in self-reported digital competencies were observed across all three implemented modules following the intervention, with large effect sizes recorded for digital innovation and change management (d = 1.04), communication and collaboration (d = 1.28), and data management and digital literacy (d = 1.30). At the item level, the largest gains were observed for cultural competence (d = 0.86), adaptive communication (d = 0.85), and data protection (d = 0.82), while competencies requiring sustained interpersonal practice, such as conflict resolution (d = 0.33), showed smaller but still significant improvements, suggesting that the curriculum’s structured delivery was most effective for directly taught competencies.

The absence of a strong association between perceived improvement and objective score change emphasizes the complexity of self-assessment in digital learning contexts, while the positive relationship between course satisfaction and performance underscores the role of engagement in professional education. Ceiling-effect findings suggest that participants with lower baseline self-reported competencies showed greater observed gains. However, these patterns should be interpreted cautiously in light of the small number of non-completers and the exploratory nature of the attrition analysis.

Overall, these findings support the feasibility and promise of the Romanian H-PASS training model for strengthening digital competencies within the healthcare workforce, while controlled studies are needed to confirm effectiveness more rigorously. Future research will extend these findings through longitudinal follow-up and controlled study designs to evaluate sustained behavioural change and long-term integration of digital skills into clinical practice.

## Figures and Tables

**Table 1:** Demographic characteristics of pharmacists included in the final analytical sample (*n* = 84) from the Romanian pilot of the H-PASS blended digital health training programme, Bihor County, Romania, May–July 2025.

Characteristic	Category	*n*	%
Gender	Female	75	89.3
	Male	9	10.7
Age Group	18–24	1	1.2
	25–34	26	31.0
	35–44	26	31.0
	45–54	28	33.3
	55–64	3	3.6
Experience	<10 years	29	34.5
	11–20 years	35	41.7
	>20 years	20	23.8
Education	Bachelor’s	72	85.7
	Master’s	2	2.4
	Doctorate	10	11.9
Location	Urban	68	81.0
	Rural	11	13.1
	Not recorded	5	5.9

## Data Availability

The raw data supporting the conclusions of this article will be made available by the authors on request.

## Supplementary Materials

The following supporting information can be downloaded at: https://www.mdpi.com/article/10.3390/pharmacy14030064/s1, Table S1: Mapping of self-assessment survey items to competency domains within the European Health Professionals’ and the DigitAl Team SkillS Advancement (H-PASS) programme (implemented modules only). Table S2: Overview of implemented modules within the European Health Professionals’ and the DigitAl Team SkillS Advancement (H-PASS) programme and their corresponding delivery frameworks. Table S3: Post-training course evaluation items included in the analysis of the H-PASS (European Health Professionals’ and the DigitAl Team SkillS Advancement) training programme. Figure S1. Q–Q plots of paired pre–post difference scores across modules and overall composite score (*n* = 84); Table S4. Wilcoxon signed-rank tests for module- and item-level pre–post comparisons confirming robustness of inferential results; Figure S2. Cronbach’s alpha values before and after the intervention for the three curriculum-defined module scores. Baseline internal consistency was low for all modules, including a negative alpha for Module 3, and improved modestly after training. Values should be interpreted cautiously because the module groupings were defined by curriculum structure and included a limited number of items; Figure S3. Heatmap of moderator effect sizes. Impact of demographic variables on changes in self-reported competency scores. Values displayed as 0.000 represent effect sizes below the rounding threshold (<0.0005) and should be interpreted as negligible rather than exact zero effects. The low color intensity and non-significant *p*-values indicate that training outcomes were consistent across all subgroups.

## Abbreviations

The following abbreviations are used in this manuscript:

H-PASS	European Health Professionals’ and the DigitAl team SkillS advancement
EU	European Union
