# Supplementary material for: Blended Learning to Enhance Competencies Among Practicing Pharmacists: A Pre–Post Evaluation of the European Health Professionals’ and the DigitAl Team SkillS Advancement Project in Romania
Source: Pharmacy (Basel). 2026 Apr 24;14(3):64. doi: 10.3390/pharmacy14030064 (PMC13214688; doi:10.3390/pharmacy14030064)
Supplement: Supplementary file 1 [file pharmacy-14-00064-s001.zip › pharmacy-4227734-supplementary.pdf]

Supplementary Materials

Table S1. Mapping of self-assessment survey items to competency domains within the European Health Professionals' and the DigitAl Team SkillS Advancement (H-PASS) programme (implemented modules only).

Local module (Results)	H-PASS module	Survey item code	Self-assessment statement (Likert 1–5)	H-PASS component	LeO type*
1	1	m1i2	I regularly look for digital solutions for my everyday challenges.	Digital wellbeing	skill
1	1	m1i3	I am open to experimenting with new digital tools in my work environment.	Digital wellbeing	attitude
1	1	m1i5	I feel confident in planning and delivering change processes effectively in my work.	Digital technologies in healthcare	skill
1	1	m1i6	I am open to modify my own practices when implementing new technologies or processes.	Digital technologies in healthcare	attitude
2	2	m2i2	I feel confident communicating with different partners in the healthcare setting both face-to-face and online.	Communication	skill
2	2	m2i3	For me it's important to adjust my communication style to my communication partner's needs.	Communication	attitude
2	2	m2i5	I feel competent working in teams in both face-to-face and online settings.	Team	skill
2	2	m2i6	I promote collaboration of partners of different backgrounds in healthcare teams.	Team	attitude
2	2	m2i8	I always succeed to resolve conflicts within my team to maintain a positive work environment.	Conflict	skill
2	2	m2i9	I am proactive in conflict resolution in my work setting.	Conflict	attitude
2	2	m2i11	I feel competent in adjusting healthcare processes or care plans to respect the cultural preferences of patients or colleagues.	Culture	skill
2	2	m2i12	I am open to change my behaviour to respect cultural differences.	Culture	attitude
3	4	m4i2	I feel confident in using online and digital sources to search, filter, and manage healthcare data.	Data in the Healthcare system	skill

Local module (Results)	H-PASS module	Survey item code	Self-assessment statement (Likert 1–5)	H-PASS component	LeO type*
3	4	m4i3	I am conscious about choosing the sources of data and information I use.	Data in the Healthcare system	attitude
3	4	m4i5	I feel competent in protecting personal and sensitive data as well as my digital wellbeing in my daily work.	Safety	skill
3	4	m4i6	I am actively watching out for online threats in my daily work.	Safety	attitude
3	4	m4i8	I feel competent in using digital tools to manage and present healthcare data to support decisions.	Working with Data	skill
3	4	m4i9	I am consciously assessing data for possible biases and inaccuracies in my daily work.	Working with Data	attitude

LeO, learning outcome.

Table S2. Overview of implemented modules within the European Health Professionals' and the DigitAl Team SkillS Advancement (H-PASS) programme and their corresponding delivery frameworks.

Local module (Results)	H-PASS module	Module title (H-PASS)	Core components covered (per H-PASS)	Delivery framework (as implemented/designed)
1	1	Leading digital transformation	Digital wellbeing; Digital technologies in healthcare; Leading digital change	Blended learning combining asynchronous e-learning (interactive lexical content with exercises; individual assignments) with synchronous trainer-facilitated onsite sessions (case-based activities).
2	2	Communication, teams, and culture in digital times	Communication; Team; Conflict; Culture	Blended learning combining asynchronous e-learning (interactive lexical content with exercises; individual and group assignments) with synchronous trainer-facilitated onsite sessions (discussion/role-play/case-based activities).
3	4	Data in healthcare	Data in the Healthcare system; Safety; Working with Data	Blended learning combining asynchronous e-learning (interactive lexical content with exercises; individual and group assignments) with synchronous trainer-facilitated onsite sessions (simulation/case-based discussion).

Table S3. Post-training course evaluation items included in the analysis of the H-PASS (European Health Professionals' and the DigitAl Team SkillS Advancement) training programme.

Survey item code	Evaluation statement	Construct assessed	Response scale
E1	How satisfied were you with the H-PASS training overall?	Overall training satisfaction	Likert 1–5 (Very dissatisfied – Very satisfied)
E2	How well do you feel the content covered in the training is applicable to your daily work?	Perceived applicability to professional practice	Likert 1–5 (Not applicable at all – Highly applicable)
E3	How much do you feel you have improved your digital skills in this training? (DigComp framework)	Perceived digital skill improvement	Ordinal scale 1–5 (Not at all – Greatly)
E7	Do you feel more confident in teamwork skills in digital settings after completing this training?	Digital teamwork confidence	Likert 1–5 (Not at all – A great deal)

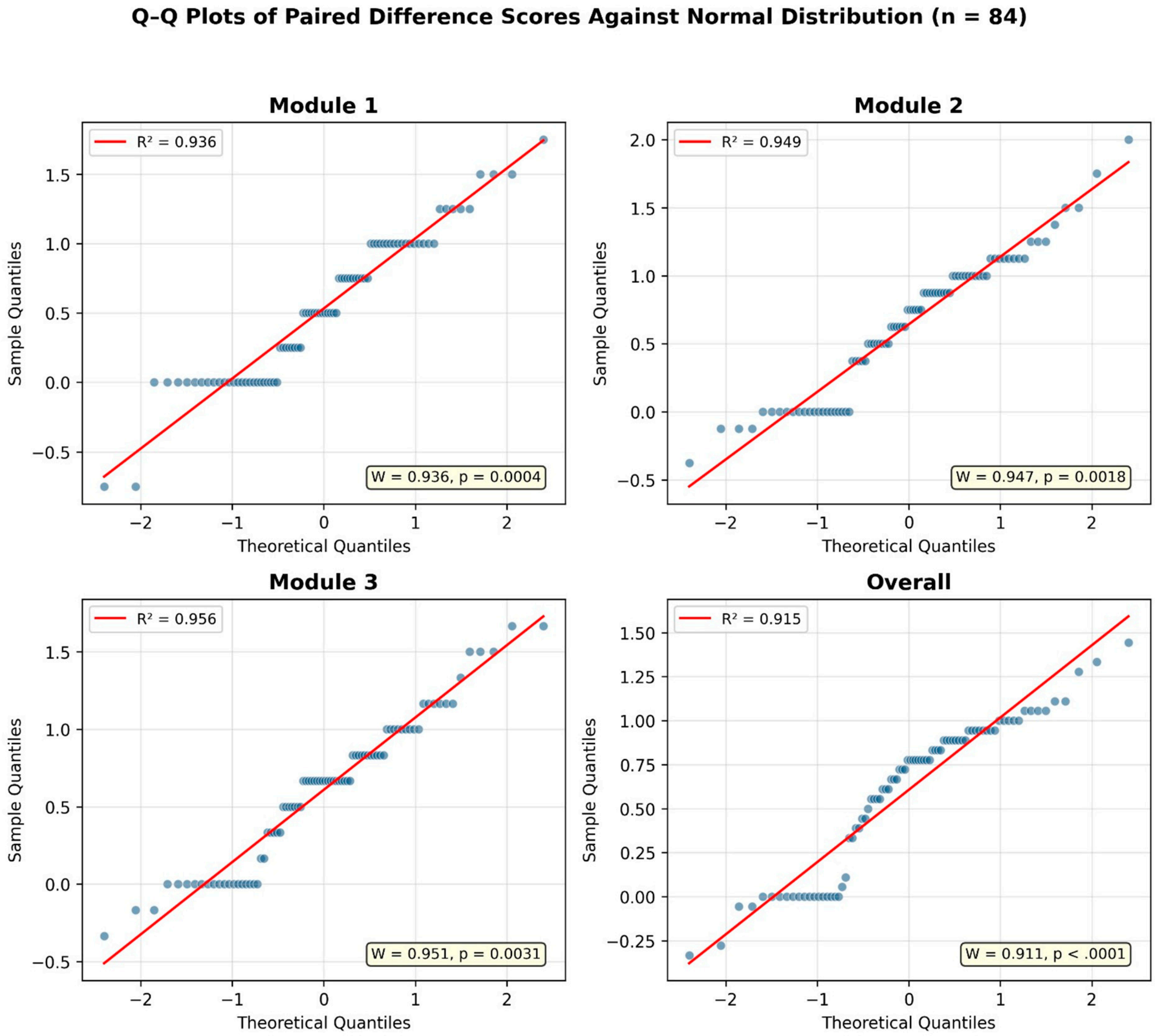

Figure S1. Q–Q plots of paired pre–post difference scores across modules and overall composite score (n = 84).

Table S4. Wilcoxon signed-rank tests for module- and item-level pre–post comparisons confirming robustness of inferential results.

Variable	Type	n	Mean Change	SD	Shapiro-Wilk W	Shapiro-Wilk p	t-statistic	t-test p	Wilcoxon W	Wilcoxon p	Cohen's d	Concordance
Module 1	Module	84	0.533	0.513	0.936	0.000400827	9.517	6.04E-15	56	2.03E-10	1.038	Yes
Module 2	Module	84	0.643	0.501	0.947	0.001762847	11.765	2.27E-19	12.5	2.67E-12	1.284	Yes
Module 3	Module	84	0.609	0.469	0.951	0.00313885	11.906	1.21E-19	12.5	1.64E-12	1.299	Yes
Overall	Overall	84	0.607	0.422	0.911	2.34E-05	13.191	4.50E-22	16	1.01E-12	1.439	Yes
m1i2	Item	84	0.679	0.894	0.872	5.88E-07	6.957	7.39E-10	133	4.05E-08	0.759	Yes
m1i3	Item	84	0.524	0.885	0.871	5.46E-07	5.427	5.56E-07	166.5	2.90E-06	0.592	Yes
m1i5	Item	84	0.298	0.655	0.692	5.49E-12	4.167	7.52E-05	30	0.000162392	0.455	Yes
m1i6	Item	84	0.631	0.757	0.797	2.14E-09	7.639	3.38E-11	18	2.80E-09	0.834	Yes
m2i2	Item	84	0.726	0.91	0.874	6.60E-07	7.315	1.47E-10	64	1.54E-08	0.798	Yes
m2i3	Item	84	0.714	0.844	0.839	4.05E-08	7.755	1.99E-11	30	7.22E-09	0.846	Yes
m2i5	Item	84	0.595	0.893	0.869	4.30E-07	6.107	3.14E-08	96	4.08E-07	0.666	Yes
m2i6	Item	84	0.762	0.926	0.882	1.35E-06	7.537	5.38E-11	68	7.07E-09	0.822	Yes
m2i8	Item	84	0.214	0.641	0.688	4.60E-12	3.062	0.002961817	47.5	0.003445929	0.334	Yes
m2i9	Item	84	0.702	0.889	0.864	2.88E-07	7.243	2.04E-10	62	2.22E-08	0.79	Yes
m2i11	Item	84	0.774	0.896	0.884	1.69E-06	7.911	9.77E-12	78	2.08E-09	0.863	Yes
m2i12	Item	84	0.655	0.976	0.893	4.07E-06	6.152	2.59E-08	136	2.88E-07	0.671	Yes
m3i2	Item	84	0.631	0.915	0.841	4.53E-08	6.317	1.26E-08	52	2.51E-07	0.689	Yes
m3i3	Item	84	0.714	0.964	0.9	7.42E-06	6.791	1.56E-09	133	4.29E-08	0.741	Yes
m3i5	Item	84	0.75	0.917	0.864	2.92E-07	7.498	6.43E-11	46.5	9.01E-09	0.818	Yes
m3i6	Item	84	0.631	0.833	0.861	2.27E-07	6.944	7.85E-10	70	3.86E-08	0.758	Yes
m3i8	Item	84	0.607	0.822	0.837	3.34E-08	6.772	1.69E-09	46.5	7.04E-08	0.739	Yes
m3i9	Item	84	0.321	0.838	0.689	4.84E-12	3.514	0.000716611	42.5	0.000839907	0.383	Yes

SD, standard deviation; n, number of participants.

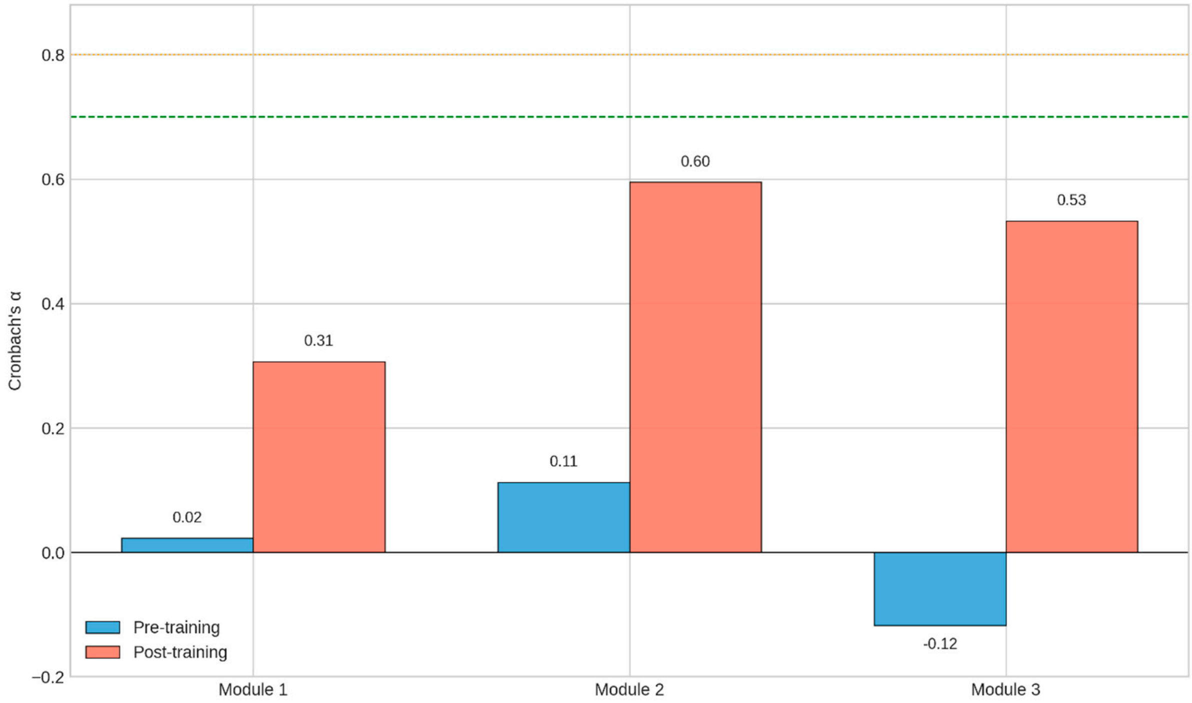

Figure S2. Cronbach's alpha values before and after the intervention for the three curriculum-defined module scores. Baseline internal consistency was low for all modules, including a negative alpha for Module 3, and improved modestly after training. Values should be interpreted cautiously because the module groupings were defined by curriculum structure and included a limited number of items.

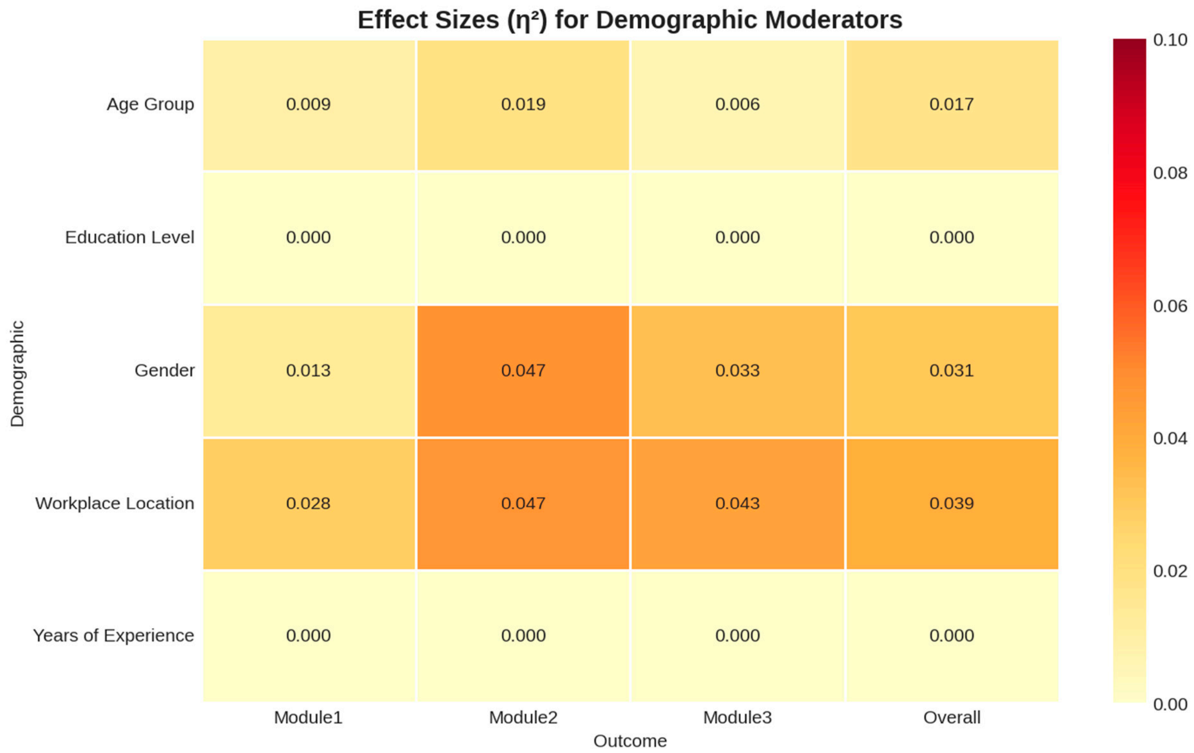

Figure S3. Heatmap of moderator effect sizes. Impact of demographic variables on changes in self-reported competency scores. Values displayed as 0.000 represent effect sizes below the rounding threshold (< 0.0005) and should be interpreted as negligible rather than exact zero effects. The low color intensity and non-significant p-values indicate that training outcomes were consistent across all subgroups.
